# The components of tumor microenvironment as biomarker for immunotherapy in metastatic renal cell carcinoma

**DOI:** 10.3389/fimmu.2023.1146738

**Published:** 2023-06-07

**Authors:** Jiaming Su, Lu Zhou, Zhe Zhang, Xue Xiao, Yanning Qin, Xiaoying Zhou, Tingting Huang

**Affiliations:** ^1^ Department of Otorhinolaryngology and Head and Neck Surgery, First Affiliated Hospital, Guangxi Medical University, Nanning, China; ^2^ Key Laboratory of Early Prevention and Treatment for Regional High Frequency Tumor (Guangxi Medical University), Ministry of Education, Nanning, China; ^3^ Guangxi Medical University, Nanning, China; ^4^ Life Science Institute, Guangxi Medical University, Nanning, China; ^5^ Department of Radiation Oncology, First Affiliated Hospital of Guangxi Medical University, Nanning, China

**Keywords:** metastatic renal cell carcinoma, immunotherapy, tumor microenvironment, biomarker, PD-L1

## Abstract

Substantial improvement in prognosis among metastatic renal cell carcinoma (mRCC) patients has been achieved, owing to the rapid development and utilization of immunotherapy. In particular, immune checkpoint inhibitors (ICIs) have been considered the backbone of systemic therapy for patients with mRCC alongside multi-targeted tyrosine kinase inhibitors (TKIs) in the latest clinical practice guidelines. However, controversies and challenges in optimal individualized treatment regarding immunotherapy remains still About 2/3 of the patients presented non-response or acquired resistance to ICIs. Besides, immune-related toxicities, namely immune-related adverse events, are still elusive and life-threatening. Thus, reliable biomarkers to predict immunotherapeutic outcomes for mRCC patients are needed urgently. Tumor microenvironment (TME), consisting of immune cells, vasculature, signaling molecules, and extracellular matrix and regulates tumor immune surveillance and immunological evasion through complex interplay, plays a critical role in tumor immune escape and consequently manipulates the efficacy of immunotherapy. Various studied have identified the different TME components are significantly associated with the outcome of mRCC patients receiving immunotherapy, making them potential valuable biomarkers in therapeutic guidance. The present review aims to summarize the latest evidence on the associations between the components of TME including immune cells, cytokines and extracellular matrix, and the therapeutic responses among mRCC patients with ICI-based treatment. We further discuss the feasibility and limitation of these components as biomarkers.

## 1 Introduction

Around 430,000 newly diagnosed renal cell carcinoma (RCC) are reported yearly worldwide (1). One-fourth of these patients experience metastatic disease (mRCC) at diagnosis and another 30% develop distant metastasis after curative nephrectomy, whose estimated 5-year survival rate is only about 10-18% (2, 3). Fortunately, substantial improvement in prognosis among mRCC patients has been achieved, owing to the rapid development and revolutionized utilization of immunotherapy over the past decade (4–6). In particular, immune checkpoint inhibitors (ICIs) have been considered the backbone of systemic therapy for patients with mRCC alongside multi-targeted tyrosine kinase inhibitors (TKIs) in the latest clinical practice guidelines from the American Society of Clinical Oncology and the European Association of Urology (7, 8). However, there remain controversies and challenges in optimal individualized treatment regarding immunotherapy. Although about 1/3 of the patients experienced objective and durable responses, the majority of the patients did not benefit, presenting non-response or acquired resistance to ICIs (9). Additionally, immune-related toxicities, namely immune-related adverse events, are still elusive and can be life-threatening (10). Around 60% of the patients administrated ICIs experienced grade 3-4 treatment-related adverse events, leading to 7%-33% treatment discontinuation (11–14).

Therefore, seeking reliable biomarkers is crucial for monitoring the therapeutic efficacy and predicting the responses of immunotherapy among mRCC patients, which may be a solution to optimize the outcomes of immunotherapy-based treatment.

**Figure 1:**
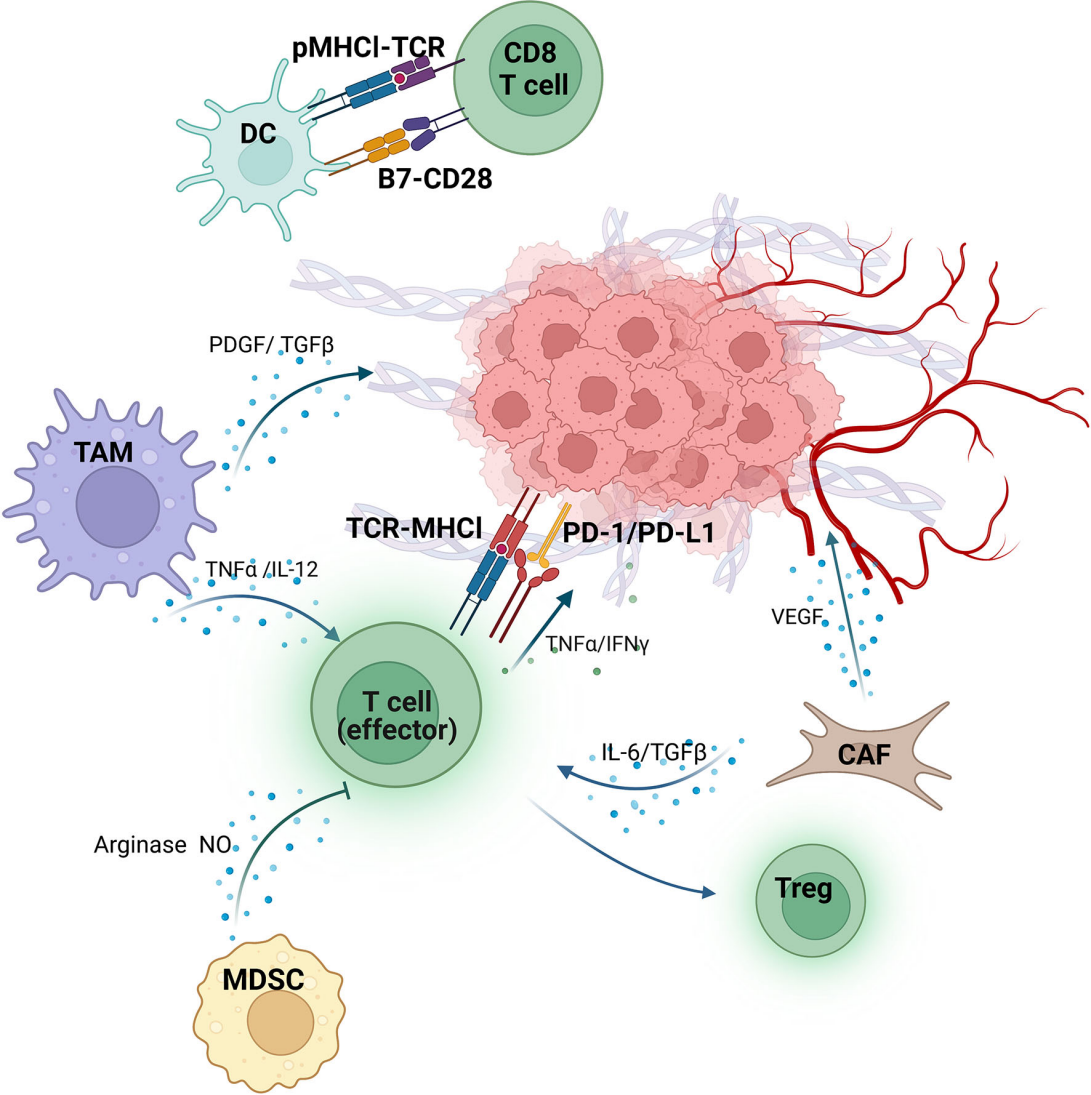
Tumor immunity/antitumor immunity in the TME of RCC. Dendritic cells (DC) present peptide-MHCI (pMHCI) complex to CD8+T cells. B7, a costimulatory molecule expressed on the surface of DC, binds to CD28 and subsequently activates CD8+T cells. The tumor-associated macrophages (TAM) secrete either platelet-derived growth factor (PDGF) and transforming growth factor β (TGF-β) to promote tumor angiogenesis, or tumor necrosis factor α (TNF-α) and IL-12 to enhance the killing function of effector T cells (T-eff). On the other hand, the function of T-eff is inhibited by Myeloid-derived suppressor cells (MDSC) via producing arginase and nitric oxide (NO). The IL-6 and TGF-β from Cancer-associated fibroblasts (CAF) lead to the transformation of T-eff into regulatory T cells (Treg), and release vascular endothelial growth factor (VEGF) to promote extracellular matrix deformation and angiogenesis as well. T-eff recognizes and kills tumor cells by binding the TCR on their surface to MHC-I on the surface of tumor cells. However, PD-L1 expressed by tumors interacts with PD-1 on T-eff significantly block their activity. Despite this, T-eff still plays an antitumor role by secreting TNF-α and interferon-γ (INFγ). (Figure was created with BioRender.com).

Recently, various studies indicate that tumor microenvironment (TME) plays a critical role in anti-tumor immunity and consequently affects the sensitivity to immunotherapy (15–18). TME consists of immune cells, vasculature, signaling molecules, and extracellular matrix and regulates tumor immune surveillance and immunological evasion through complex interplay (**Figure 1**) (19). Several investigations have revealed the potential of TME components of TME as biomarkers for immunotherapy in various solid tumors, including RCC (11, 20–22). For instance, the expression of Programmed cell death-ligand 1 (PD-L1) in mRCC has been extensively studied as a potential biomarker for immunotherapy (23–25). Besides, researches on cytokines (such as IL-8 and IL-6) and extracellular matrices have shown their potential as biomarkers for mRCC (26–29). Although intriguing progress has been made, no consensus on a reliable and feasible biomarker for immunotherapy among mRCC patients to date.

The present review aims to summarize the latest evidence on the association between key components of TME (including PD-L1 and other immune checkpoints, tumor-infiltrating T cells and T cell receptors, cytokines, and extracellular matrix) and the therapeutic responses among mRCC patients with ICI-based treatment and discuss the potential and feasibility of these components as a biomarker for mRCC patients receiving immunotherapy.

## 2 Immunotherapy for metastatic renal cell carcinoma

Along with the growing understanding of the immune responses ofRCC and considerable results achieved in numerous solid tumors by applying ICIs, medicines targeting the programmed death-1 (PD-1)/programmed death-ligand 1 (PD-L1) axis and cytotoxic T-lymphocyte associated antigen (CTLA-4) pathways have been introduced to mRCC patients since 2014 (30, 31) and received the United States Food and Drug Administration approval in 2018 (32). Furthermore, after encouraging evidence reported by a series of milestone clinical trials (9, 12, 14, 33–36), the combination of TKI and ICI was formally recommended for mRCC patients as first-line protocols by the U.S. National Comprehensive Cancer Network clinical guidelines for kidney cancer, since version -3.2022 (37–39). A brief summary of clinical practices for mRCC patients based on NCCN guideline and the results of selected crucial trials are described in **Table 1** and **Supplementary Table 1**. Although an ameliorative advancement has been yielded, a substantial amount of mRCC patients did not benefit from ICI-based therapy (40). Therefore, identifying reliable biomarkers is believed to facilitate further improvement in prolonged survival and minimize toxicities for mRCC patients (41–43).

**Table 1:** Brief summary of the NCCN guidelines (Version 3. 2022) for metastatic renal cell carcinoma.

Frist-line Therapy For Metastatic Renal Cell Carcinoma				
Risk	Preferred Regimens	Other Recommended Regimens		Useful in Certain Circumstances Treatments
Favorable-Risk Group	Axitinib+ Pembrolizumab Cabozantinib+Nivolumab Lenvatinib+Pembrolizumab	Axitinib+ Avelumab Cabozantinib Ipilimumab+ Nivolumab Pazopanib Sunitinib		Axitinib High-Dose IL-2
Intermediate-Risk Group	Axitinib+ Pembrolizumab Cabozantinib+Nivolumab Lenvatinib+Pembrolizumab Ipilimumab+ Nivolumab Cabozantinib	Axitinib+ Avelumab Pazopanib Sunitinib		Axitinib High-Dose IL-2 Temsirolimus
Poor-Risk Group	Axitinib+ Pembrolizumab Cabozantinib+Nivolumab Lenvatinib+Pembrolizumab Ipilimumab +Nivolumab Cabozantinib	Axitinib+ Avelumab Pazopanib Sunitinib		Axitinib High-Dose IL-2 Temsirolimus

Subsequent Therapy For Metastatic Renal Cell Carcinoma				
Preferred Regimens	Other Recommended Regimens		Useful in Certain Circumstances Treatments	
Cabozantinib Lenvatinib+Everolimus Nivolumab	Axitinib Axitinib+ Pembrolizumab Cabozantinib+Nivolumab Ipilimumab+Nivolumab Lenvatinib+Pembrolizumab Pazopanib Sunitinib Tivozanib Axitinib+Avelumab		Everolimus Bevacizumab High-Dose IL-2 Sorafenib Temsirolimus	

## 3 TMEs as a biomarker for immunotherapy in mRCC

TME is the cellular niche surrounding tumor cells, which consists of immune cells, stromal cells, endothelial cells, extracellular matrix (ECM), neuroendocrine cells, adipose cells, and structural molecules, forming a spatially organized, dynamic, and functional network (15, 44, 45). Recent advances suggest that TME may be one of the pivotal players in modulating tumor immune surveillance and immunological evasion, as well as regulating the response to immunotherapy (15, 41, 46). Besides, the majority of RCC, especially clear-cell RCC (ccRCC) accounting for > 70% of tumors, is regarded as immunogenic cancer, featured by infiltration of leukocytes (natural killer cells, CD8^+^ T cells, and CD4^+^ T cells) and myeloid cells (macrophages and neutrophils) as surrounding microenvironment (41, 45). Furthermore, transcriptomic studies based on The Cancer Genome Atlas (TCGA) database addressed that ccRCC were highly CD8^+^ T cell infiltrated (only 27% of the tumor showed non-infiltrated features) and presented the highest scores on both the immune infiltration and T cell infiltration among 19 cancer types (23, 24). Thus, several key components of TME has been considered a candidate for the biomarker for immunotherapy in mRCC patients (28, 47–52).

### 3.1 Programmed cell death-ligand 1

Recent clinical trials administrating ICIs-based immunotherapy in mRCC patients have reported that higher PD-L1 expression was associated with higher objective response rates (ORRs) (47, 53–55). In the PD-L1 positive population (expression ≥1%), mRCC patients treated with ICIs presented improvement in progression-free survival (PFS) or overall survival (OS) compared with those with sunitinib monotherapy (47, 53). Notably, conflict results were addressed in other trials comparing ICIs and sunitinib, where PD-L1 expression showed no predictive value in mRCC patients (12, 36, 56) (**Table 2**).

**Table 2:** Subgroup analysis of PD-L1 expression in trials of ICIs-based immunotherapy.

Trail	Assay	PD-L1 exp.	PFS HR (95%CI)	OS HR (95%CI)	Predictive value	Refs
Atezolizumab plus bevacizumab vs sunitinib	Ventana PD-L1 (SP142)	<1%	0·93(0·75–1·15)	–	gradient of increasing PFS benefit with increasing PD-Ll expression	(47)
		1%-4%	0·78(0·57–1·06)			
		5%-9%	0·69(0·38–1·22)			
		≥10%	0·56(0·26–1·19)			
Pembrolizumab plus Axitinib vs Sunitinib	IHC 22C3 pharmDx	<1%	0.87 (0.62-1.23)	0.59 (0.34-1.03)	not recommended as a biomarker	(56)
		≥1%	0.62 (0.47-0.80)	0.54 (0.35-0.84)		
Avelumab plus Axitinib vs Sunitinib	Ventana PD-L1 (SP263)	<1%	0.83 (0.60-1.16)	0.72 (0.45-1.17)	not recommended as a biomarker	(12)
		≥1%	0.64 (0.51-0.80)	0.85 (0.61-1.8)		
Lenvatinib plus Pembrolizumab vs Sunitinib	IHC 22C3 pharmDx	<1%	0.39 (0.26–0.59)	–	not recommended as a biomarker	(57)
		≥1%	0.40 (0.27–0.58)			
Nivolumab plus Cabozantinib vs Sunitinib	Dako PD-L1 IHC 28–8 pharmDx	<1%	0.52(0.40-0.67)	0.51(0.34-0.75)	OS benefit was uncertain	(36)
		≥1%	0.49(0.32-0.73)	0.80(0.48-1.34)		
Nivolumab plus Ipilimumab vs Sunitinib	Dako PD-L1 IHC 28-8 pharmDx	<1%	1.00(0.80-1.26)	0.73(0.56-0.96)	not recommended as a biomarker	(53)
		≥1%	0.46(0.31-0.67)	0.45(0.29-0.71)		

HR, hazard ratio; CI, confidence interval; PD-L1, programmed cell death-ligand 1; exp, expression; Refs, References; OS, overall survival; PFS, progression-free survival.

Currently, several studies have considered different measurements for PD-L1-based evaluation among mRCC patients with ICI-based treatment. Chen et al. reported that patients with PD-L1^+^ circulating tumor cells (CTCs) experienced higher disease control rates than others (73%, 11/15 vs. 20%, 1/5). After treatment, 82% of patients with controlled diseases (9/11) presented a decrease in the expression of PD-L1^+^ CTCs, whereas all the patients with progressive diseases (100%, 4/4) showed an increased or stable expression of PD-L1^+^ CTCs (58). In addition, Incorvaia and colleagues demonstrated that patients with higher baseline plasma soluble PD-L1 concentrations (> 0.66 ng/ml) might associate with longer PFS after nivolumab treatment (*p* < 0.0001) (59).Moreover, the combination of PD-L1 expression with other biomarkers is under investigation and expected to improve the predictability of immunotherapy. Among mRCC patients treated with nivolumab, those with high tumor cells PD-L1 expression and a high percentage of CD8^+^ PD-1^+^ TIM-3^-^ LAG-3^-^ tumor-infiltrating cells experienced longer immune-related PFS and better immune-related ORR (60). Chouaib and coworkers reported that the expression status of AXL (receptor tyrosine kinases) was closely associated with PD-L1 status, especially in the population with tumor-suppressor gene von Hippel-Lindau (VHL) inactivation (61). They further revealed that patients with accompanied high AXL expression and PD-L1 expression had worst survival than others (61).

However, these evidences mentioned above are insufficient to support if the expression of PD-L1 in tumor tissue alone served as a reliable biomarker. Firstly, lack of a standardized detection method, consistent thresholds of PD-L1 positivity, and consensus on how to score the expression level of PD-L1 in tumor cells only or including immune cells in TME in these studies (62). Secondly, conducting and interpreting the immunohistochemical results is usually subject to standardized guidelines and pathologists’ experience (63). Different immunohistochemical protocols were applied to measure the PD-L1 expression, leading to a difficulty in comparing the results and result interpretation directly. Thirdly, the different histopathological features of RCC and TME may affect the association between PD-L1 expression and treatment outcomes. For example, sarcomatoid and rhabdoid features increased CD8+ T-cell infiltration and up-regulated PD-L1 expression, showing higher response rate ICIs (64). Fourthly, PD-L1 expression was detected in various portions of study subjects, and its association with treatment outcome was analyzed in a subgroup with limited power, leading to failed comparisons among studies. Therefore, researches who had aware those difficulties suggested that the detection of circulating PD-L1 levels might complement the IHC-based measurement of PD-L1 expression in tissue based on experimental but encouraging results (partially presented above). In addition, the single biomarker might not be enough, but establishing a predictive model, a combination with other biomarkers would be a better strategy to improve the accuracy.

### 3.2 Other immune checkpoints

Beyond PD-1/PD-L1, the expression of cytotoxic T-lymphocyte-associated Protein 4 (CTLA-4) in RCC tumor-infiltrating monocytes was identified as an independent prognostic factor in RCC patients and significantly associated with worse outcome (65). The overexpression of CTLA4 due to promoter hypomethylation in RCC patients treated with ICI might be an independent factor for favorable outcomes (PFS: HR 1.94 (95% CI 1.09 to 3.44), *p* = 0.024; OS: HR 2.14 (95% CI 1.01 to 4.57), *p* = 0.048) (49). Besides, Lymphocyte activation gene-3 (LAG-3) and T cell immunoglobulin and mucin-containing molecule 3 (TIM-3) have been found to be associated with poor prognosis among mRCC patients (66, 67). Notably, the evidence on the roles of the immune checkpoints other than PD-1/PD-L1 in mRCC patients with immunotherapy (targeting PD-1/PD-L1 axis) is limited. Their roles in other immunotherapy are under investigation and related results are awaited.

### 3.3 Tumor-infiltrating T cells and T cell receptors

The abundance and composition of TITCs are valuable for prognostic prediction of cancer patients (68). Increased immune infiltration has been observed in RCC patients with nivolumab administration (69). A positive associations was found between the improved therapeutic response of nivolumab in ccRCC patients and the abundance of T-cell subsets in biopsies collected at baseline (*p* = 0.03) and day 28 (*p* < 0.01) of treatment (50). Detailed study of the T cell population may deepen the understanding of TITCs’ role in the immunotherapy of mRCC. Scientists found that patients with raised tumor-infiltrating CD8^+^ T cells and TCF-1^+^stem cell-like CD8^+^ T cells at the time of surgery tend to experienced robust immune responses and improved survival after subsequent immunotherapy (51). Those with larger expansions of HLA-DR^+^/CD38^+^/CD8^+^ T cells in peripheral blood after one cycle of immunotherapy had more significant tumor shrinkage (p < 0.05) and longer PFS (*p* = 0.006). Similarly, a higher TNFRSF9 CD8^+^ T cells infiltration was addressed to be associated with greater reduction of tumor size (*p* = 0.003) and better PFS (*p* = 0.012) in ccRCC patients receiving nivolumab (70). However, some non-responders can also present high T-cell infiltration (50). In addition, infiltration of some subsets of CD8 T cells such as PD-1^+^TIM-3^+^CD8^+^, CXCL13^+^CD8^+^, and CD39^+^CD8^+^ were associated with poor prognosis in RCC without immunotherapy (71–73). It is notable that CD8+T cells are activated and eventually differentiate into a phenotypically depleted terminal state in responders, according to single-cell transcriptome analysis of advanced RCC before and after ICI treatment (74). The expression of checkpoint molecules and anti-inflammatory signals was increased. It should be recognized that antitumor immunity is a dynamic process, and the composition and potential function of TITCs in different stages of immunotherapy will change accordingly.

On the other hand, TCR repertoire has been considered as a candidate biomarker for therapeutic monitoring and prognostic evaluation in cancer patients (75, 76). In the analysis of the peripheral TCR of HLA-DR^+^ CD38^+^ CD8 T cells, newer additional TCR clonotypes emerge in patients with a clinical benefit after one cycle of treatment (51). In a phase II study of nivolumab-treated mRCC patients, researchers conducted TCR analysis and found a higher pre-treatment expanded TCR clonality in ICI responders than non-responders (*p* = 0.042) (25). After treatment, expanded TCR clones in ICI responders were more likely to maintain than in non-responders whose TCR clones were usually replaced (*p* = 0.024). Maintaining similar TCR clones in tumor tissue after treatment was correlated with therapeutic response, but no correlation was found in peripheral TCR clones (25). Similarly, Kato et al. observed that expanded TCR clones preexisted in responders’ circulation before immunotherapy and maintained a long-term antitumor immune response after treatment (77). However, contradicting the former study, they found that patients with increased peripheral TCR clones after treatment had better OS and PFS than those with decreased peripheral TCR clones (*p* = 0.044 and *p* = 0.028, respectively) (77).

The presented studies yielded inconsistent associations between TITCs/TCR repertoire and ICI-based therapeutic outcomes. We are aware that our understanding of the diversity of the T cell population and the complexity of their functions is, to date, the tip of the proverbial iceberg.

### 3.4 Cytokines

Cytokines are involved in the physiological and pathological processes of mRCC patients, as well as the efficacy of treatment. mRCC patients undergoing ICI-based treatment with lower baseline of IL-8 has higher ORR (*p* = 0.047) (26). Elevated IL-8 expression might induce epithelial-mesenchymal transformation (EMT) and promote distant metastasis (78). Two comprehensive studies based on clinical trials underlined that circulating IL-8 might be a potential prognostic biomarker for patients administrating ICIs. Schalper and colleagues analyzed the data from four trials of patients with various cancers (advanced RCC, melanoma, and non-small-cell lung cancer) (52). They revealed that higher levels of pretreatment serum IL-8 were associated with shorter OS [HR = 2.56 (95% CI 1.89-3.54)] and lower PFS [HR = 1.36 (95% CI 1.07-1.72)] (52). Additionally, higher serum IL-8 before treatment was associated with poorer survival across cancer types, regardless of treatment strategies. In another study looking at ICIs in managing patients with advanced RCC and urothelial carcinoma, a significantly negative association between baseline plasma IL-8 levels and treatment outcomes is demonstrated, which is similar to the former finding (79). Except for IL-8, an analysis of studies in patients with metastatic renal cell carcinoma treated with IFN-α and Bevacizumab showed that IL-6 and hepatocyte growth factor (HGF) were associated with shorter OS [IL-6: HR = 1.27, 95% CI (1.11-1.42); HGF: HR = 1.19; 95% CI (1.00-1.33)] (29). Sang et al. found that among mRCC patients receive treatment with Pembrolizumab plus Axitinib, the shorter PFS [HR = 3.51, 95% CI (1.54-7.98), p = 0.003] and worse OS [HR = 7.18, 95% CI (2.26-22.82), p = 0.001] were found in those patient with higher IL-6, patients with high IL-6 had worse OS than those with low IL-6 (80). Although further validation is required, these promising results highlight the possibility of some cytokines as reliable and easily measurable predictive biomarkers for RCC and other solid tumor upon ICI treatment (81).However, limited information can be captured by a single measurement of serum cytokine at a single time point (82, 83). Besides, cytokines could be affected by numerous diseases beyond tumors (84–86). Therefore, the specificity of cytokines is relatively low, which obstacles its application as a biomarker.

### 3.5 Extracellular matrix

Substantial alterations of ECM around solid tumor contribute tremendously in the invasion of tumor cells, thereby initiating metastasis (87, 88). A series of investigations have focused on the components of ECM in RCC patients with metastasis and their treatment responses (27, 28, 89–91). For example, higher levels of transmembrane collagen COL23A1, a ligan of integrin α2β1 (92) [HR = 3.024, 95% CI (1.22-7.49)] and hyaluronan, a high molecular weight unbranched polysaccharide [HR = 1.4; 95% CI (1.02-2.0)] were associated with shorter survival CD248, being identified to localize to the stromal compartment in cancers, serves a key role in myofibroblast generation and accumulation (93). mRCC patients with CD248 overexpression and cancer-associated fibroblasts (CAF) infiltration were experienced poorer 5-year OS (58.3%) comparing those with low infiltration (27); Feng et al. reported a higher level of SPARC-related modular calcium-binding (SMOC2), which promotes matrix assembly and stimulate angiogenic activity was associated with worse 5-year survival rates than patients with lower SMOC2 (64% and 79%, respectively) (28). These findings have not yet been validated in prospective studies with sufficient subjects. More data is awaited to qualify the availability of ECM as a candidate biomarker for immunotherapy. On the other hand, evaluating the expression levels of particular components in ECM has been a practically challenge due to all of these components are expressed and function normally among adjacent healthy epithelial cells as well (94).

## 4 Future perspectives

Over the past decade, the utility of ICI-based systemic therapy has been attributed to the substantial improvement in prognosis among mRCC patients. To further improve clinical efficacy, and early identification of response and non-response in mRCC patients receiving immunotherapy to reduce the cost of treatment and avoid the damage of immune-related adverse reactions, seeking reliable biomarkers is crucial to predict the outcome and monitor therapeutic management.

As the most extensively studied component in TME, PD-L1 expression has not yet been available in monitoring treatment strategy and predicting outcomes due to inconsistent results across cancer types and ICI combinations. Developing a standardized, interchangeable detection assay and defining a uniform threshold of positivity of PD-L1 expression might improve the comparability across studies and practicality in clinical management. In addition, complementary circulating PD-L1 measurements and a combination of PD-L1 and other biomarkers might accelerate the application of PD-L1 to the bedside. Moreover, fully elucidating how distinct histopathological features, TME signatures, and patient characteristics affect the expression of PD-L1 and its relation with ICI-based treatment outcomes could facilitate the progress of seeking reliable biomarkers. Last but not least, any promising results that have been announced should be further validated in well-designed prospective studies with robust power.

In addition, encouraging but inconsistent results of TITCs and TCR as predictive markers for ICI-based treatment among mRCC patients were yielded from previous research. A more particular knowledge of the dynamics of the T cell population in response to immunotherapy among RCC patients is warranted to monitor therapeutic decisions better (95, 96). Comprehensive characterization of immune cell phenotypes and their interrelationships in the tumor microenvironment can promote immune cells such as T cells to become biomarkers and drug targets. Using mass spectrometry, a method that can be used to analyze large numbers of cells, Chevrier et al. identified 17 tumor-associated macrophage phenotypes, 22 T-cell phenotypes, and a unique immune composition associated with progression-free survival in ccRCC (97). The development of single-cell transcriptome analysis and mass spectrometry have revealed the complex network of immune cells in the tumor microenvironment, which is expected to help us better understand the therapeutic response in the context of immunotherapy. TCR repertoire analysis based on deep sequencing provides a reliable assessment to reveal the clonal richness and diversity of the T cell population, to chase the longitudinal changes of T cell clones with repeated sampling on both tissue and blood, and to measure the expansion of T cell clones. Thus, introducing TCR repertoire analysis into future investigations on immunotherapy treatment outcomes among patients with RCC and other solid tumors should facilitate the progression in clinical management.

Serum cytokine IL-8 has been suggested as a feasible biomarker for prognosis prediction of ICI-based treatment. Next step, further validation trials are warranted to confirm its capability and test its specificity and sensitivity as a biomarker. Moreover, the multi-measurement of correlated cytokines which function in a synergistic network (such as IL-8, IL-6, and TNF) from one sample might raise the value of the prediction of outcomes (98), and identification of cytokine composite signatures associated with prognosis may be an important approach to improve specificity and accuracy (99) Recent advances in ECM detection suggested that ECM derivatives measured in blood might be an attractive supplement to ECM detection (100).

## 5 Conclusions

In summary, accumulating data prompt that components of TMEs, such as PD-L1, CTLA-4, LAG-3, TIM-3, infiltrating T cell and T cell receptors, cytokines, and ECM, might be candidates of predictor for ICI-based treatment outcome among mRCC patients. However, they have yet to be served as reliable and practical biomarkers applying to the clinic immediately. Thus, future investigations in developing standardized measurement, expanding knowledge on the functional network of TMEs, and validations are warranted to overcome these issues and facilitate a continuous improvement of the clinical benefit of ICI-based treatment among mRCC patients. In addition, we believe that a comprehensive prediction model for mRCC patients by incorporating both classical prediction models (such as International Metastatic Renal-Cell Carcinoma Database Consortium criteria) and various TME components would have the potential to improve the accuracy of predictions. Furthermore, novel testing and analyzing techniques and approaches, such as TCR repertoire analysis, single-cell multi-omic analysis, multiplex label-based immunoassays, and mass spectrometry, are encouraged to apply in future studies, both in mechanism exploration and clinical settings.

## Author contributions

All authors listed have made a substantial, direct, and intellectual contribution to the work and approved it for publication.
