# Supplementary material for: The components of tumor microenvironment as biomarker for immunotherapy in metastatic renal cell carcinoma
Source: Front Immunol. 2023 Jun 7;14:1146738. doi: 10.3389/fimmu.2023.1146738 (PMC10282412; doi:10.3389/fimmu.2023.1146738)
Supplement: Supplementary file 1 [file DataSheet_1.pdf]

Supplementary Material

1 Supplementary Table

Supplementary Table 1 Crucial trials of immunotherapy

**Trial**	**N**	**Experimental Arm**	**Control Arm**	**Median OS (month)**	**Median PFS (month)**	**ORR**
CheckMate 025 (14)	803	Nivolumab	Everolimus	25.8 vs19.7	4.2 vs 4.5	22.9% vs 4.1%
CheckMate 214 (9)	1096	Nivolumab Ipilimumab	Sunitinib	55.7 vs38.4	12.3 vs 12.3	39.3%vs32.4%
CheckMate 9ER (36)	651	Nivolumab Cabozantinib	Sunitinib	NR vs NR	16.6 vs 8.3	55.7%vs27.1%
KEYNOTE-426 (97)	861	Pembrolizumab Axitinib	Sunitinib	NR vs 35.7	15.4 vs 11.1	60.4%vs39.6%
Keynote 581 (98)	1069	Pembrolizumab Lenvatinib	Sunitinib	NR vs NR	23.9 vs 9.2	71% vs 36%
		Lenvatinib Everolimus	Sunitinib	NR vs NR	14.7 vs 9.2	54% vs 36%
Javelin Renal 101 (12)	886	Avelumab Axitinib	Sunitinib	NR vs NR (ITT) NR vs 28.6 (PD-L1$^+$)	13.3 vs 8.0 (ITT) 13.8 vs 7.0 (PD-L1$^+$)	52.5%vs27.3% (ITT) 55.9%vs27.2% (PD-L1$^+$)
IMmotion150 (33)	305	Atezolizumab	Sunitinib	---	6.1 vs 8.4 (ITT) 5.5 vs 7.8 (PD-L1$^+$)	25% vs 29% (ITT) 28% vs 27% (PD-L1$^+$)
		Atezolizumab Bevacizumab	Sunitinib	---	11.7 vs 8.4 (ITT) 7.8 vs 7.8 (PD-L1$^+$)	32% vs 29% (ITT) 46% vs 27% (PD-L1$^+$)
ITT= intent-to-treat; NR=not reached; OS=overall survival; PFS=progression-free survival; ORR=objective response rate; PD-L1= programmed cell death-ligand 1						
